# Editorial: Similarities and differences between substance-related and non-substance-related addictive behaviors

**DOI:** 10.3389/fpsyg.2026.1857478

**Published:** 2026-05-07

**Authors:** Salvatore Campanella, Laura Angioletti, Michela Balconi

**Affiliations:** 1 Laboratory of Medical Psychology and Addiction, ULB Neuroscience Institute (UNI), CHU Brugmann, Université Libre de Bruxelles (U.L.B.), Brussels, Belgium; 2 International research center for Cognitive Applied Neuroscience (IrcCAN), Università Cattolica del Sacro Cuore, Milan, Italy; 3 Research Unit in Affective and Social Neuroscience, Department of Psychology, Università Cattolica del Sacro Cuore, Milan, Italy

**Keywords:** addictive behaviors, behavioral addiction, Response Inhibition, Salience Attribution, substance-related addiction

Altered inhibitory skills combined with an increased salience of addiction-related cues are the two main disturbed cognitive and emotive mechanisms related to addictive behaviors. Indeed, addictive stimuli are associated through classical conditioning stimuli to rewarding and highly motivational responses (such as excessive alcohol consumption or gambling). These stimuli, therefore, grab patients' attention and elicit conditioned responses (e.g., consumption behaviors that have become dominant over time and that cannot be regulated or stopped). The Impaired Response Inhibition and Salience Attribution (I-RISA) model, grounded in extensive neuroimaging research, has identified specific dysfunctions across six large-scale brain networks (Goldstein and Volkow, 2011), providing a framework for understanding both behavioral and substance-related addictions (Zilverstand et al., 2018). While we can clearly imagine how various substances (such as alcohol, cannabis, cocaine, heroin, and others), acting as neurotoxic agents that kill neurons (Singh and Verma, 2020), could impact various brain systems, contributing to an imbalance between (hypoactive) inhibitory and (hyperactive) attentional mechanisms, the situation is less clear when considering non-substance-related addictive behaviors (Balconi, 2021). Indeed, in the absence of any ingestion of a neurotoxic agent, how can we understand that behaviors such as playing video games, gambling, watching TV, or using social media may become “addictive,” and represent (mainly among youths) a significant public health concern? (Campanella, 2024).

Within this theoretical framework, the present Research Topic highlights the dysfunctional global similarities that suggest a common neurocognitive etiology for these disorders and identify the main differences (e.g., specific responses to rewarding cues and risk factors) that point to the specificity of substance-related and non-substance-related addictive behaviors. To this end, in order to be able to screen for various addictive behaviors, Faiad et al. and Sahin et al. worked on the validation of Brazilian and Turkish versions, respectively, of the Brief Screener for Substance and Behavioral Addiction. Other original research was devoted to the description of various altered mechanisms induced by specific substance-related or non-substance-related behaviors. For example, Dampuré et al. investigated the behavioral and neural correlates of smoking habits, Yaakoubi et al. focused on the executive and motor dysfunctions related to screen time/smartphone addiction, Duman et al. reported the different postural correlates that index different motivational stimuli, such as alcohol or food, Wang et al. highlighted the significant role of self-esteem and the teacher-student relationship in adolescent internet addiction, and Crivelli et al. compared the verbal memory dysfunctions triggered by substance use vs. gambling disorder. Interestingly, Park et al. reviewed current data concerning gaming disorder and caffeine consumption in adolescents, and Goto et al. assessed a conceptual framework of the addiction spectrum disorders. Finally, new perspectives and treatment avenues were reported by Vukadinovic, who discussed the potential clinical interest of neurofeedback in addictive disorders, while Schuerch et al. described a controlled feasibility trial for a mobile, alcohol-specific inhibition training program.

Overall, all these contributions again stressed the extreme complexity of the substance and non-substance addictive phenomena. The mechanisms behind how these habits are learned and operate, the risk factors that promote the transition from impulses to compulsive use, or the way new psychological/neurocognitive interventions may be useful at the clinical level, are still defined (among many others) as unanswered questions despite decades of research. This should naturally prompt further studies to adopt a more integrative perspective to help the field move toward improved understanding, prevention, diagnosis, and treatment (Corley et al., 2024).
